# Injection practices in 2011–2015: a review using data from the demographic and health surveys (DHS)

**DOI:** 10.1186/s12913-019-4366-9

**Published:** 2019-08-27

**Authors:** Tomoyuki Hayashi, Yvan J.-F. Hutin, Marc Bulterys, Arshad Altaf, Benedetta Allegranzi

**Affiliations:** 1 Global Hepatitis Programme, World Health Organization, Geneva, Switzerland; 2 Department of Gastroenterology, Kanazawa University and WHO Collaborating Center for Chronic Hepatitis and Liver Cancer, Kanazawa, Ishikawa Japan; 3 Integrated Service Delivery, World Health Organization, Western Pacific Region, Manila, Philippines; 4 Infection Prevention and Control, World Health Organization, Geneva, Switzerland

**Keywords:** Healthcare injection, Demographic and health surveys, DHS, Syringe, Needle, Injection practice, Injection device, Unsafe injection

## Abstract

**Background:**

Reuse of injection devices to give healthcare injections decreased from 39.8 to 5.5% between 2000 and 2010, but trends since 2011 have not been described. We reviewed results of Demographic and Health Surveys (DHS) to describe injection practices worldwide from 2011 to 2015.

**Methods:**

We searched the DHS Internet site for data published on injection practices conducted in countries from 2011 to 2015, extracted information on frequency (number of healthcare injections per person in the last 12 months) and safety (proportion of syringes and needles taken from a new, unopened package). We compared gender groups and WHO regions in terms of frequency and safety. For countries with data available, we compared injection practices 2004–2010 and 2011–2015.

**Results:**

Since 2011, 40 of 92 countries (43%) that conducted DHS surveys reported on injection practices. On average, the frequency of injection was 1.64 per person per year (from 3.84 in WHO Eastern Mediterranean region to 1.18 in WHO African region). Among those, 96.1% of injections reportedly used new injection devices (from 90.2% in the WHO Eastern Mediterranean region to 98.8% in the WHO Western Pacific region). On average, women received more injections per year (1.85) than men (1.41). Among 16 (40%) countries with data in 2004–2010 and 2011–2015, 69% improved in terms of safety. The annual number of unsafe injections reduced in 81% of countries. In Pakistan, the number of unsafe injections was the highest and did not decrease between 2006 and 2012.

**Conclusions:**

Injection practices have continued to improve in most countries worldwide, although the Eastern Mediterranean region in particular still faces unsafe practices that are not improving. Further efforts are needed to eliminate unsafe injection practices in health care settings, including through the use of reuse-prevention devices. Despite some limitations, DHS is an easily available method to measure progress over time.

## Background

A safe injection never harms the recipient, does not expose the provider to avoidable risks and does not result in any waste that is dangerous to other people [1]. The World Health Organization (WHO) estimated that in 2000, 16 billion healthcare injections were given each year in developing and transitional countries [2]. Of these, 90–95% were for therapeutic purposes, while 5–10% were immunizations [2].

Injections are often used unnecessarily when oral medicine could be equally effective [3]. WHO estimated that in 2000, 39.8% of injections were given with devices reused in the absence of sterilization [2]. Health care injections given with re-used equipment expose patients to infections with bloodborne pathogens, including hepatitis B virus (HBV) [4], hepatitis C virus (HCV) [5], and human immunodeficiency virus (HIV) [6]. Overuse of injections to administer medicines amplifies the risk of transmission. WHO estimated that in 2000, overuse and unsafe use of healthcare injections caused 30% of new infections with HBV (21 million), 41% of new infections with HCV (2 million), and 9% of new infections with HIV (260,000, annually) [7].

Since 2000, WHO worked on the establishment of policies for the safe and appropriate use of injections worldwide. The Safe Injection Global Network (SIGN) regrouped efforts from all stakeholders, including international organizations, governments, nongovernmental organization, civil society, and industry [8]. From 2004, the Demographic and Health Surveys (DHS) started to include data on injection practices for surveys conducted in several countries [9]. DHS are nationally representative population-based surveys of adult population with large sample sizes (e.g., > 5,000 households). The DHS Programme has provided technical assistance to more than 300 surveys in over 90 countries, advancing global understanding of health and population trends. New questionnaire items included injection frequency (the average number of healthcare injections reported per person per year) and injection safety (whether for the last healthcare injection, the syringe and needle came from a new, unopened package). In the year 2010, WHO commissioned an update of the 2000 injection practices estimates. This 2010 review which largely used DHS data. The results indicated that between 2000 and 2010, the proportion of reuse of injection devices dropped from 39.8 to 5.5%. Meanwhile, the frequency of use of injection to administer medications had not decreased [10]. Overuse and unsafe use of injections still led to transmission of bloodborne pathogens, although less so than in 2000. From 2000 to 2010, despite a 13% population growth, there was an estimated reduction of 87 and 83%, respectively, in the absolute numbers of HIV and HCV infections transmitted through healthcare injections. For HBV, the reduction was more marked (91%) due to the additional impact of vaccination [11].

In 2016, WHO published injection safety guidelines recommending safety engineered injection devices to eliminate unsafe injections [12]. The WHO policy document specifically addressed the use of these devices for therapeutic injections. Further, WHO launched a comprehensive behaviour change and multimodal implementation strategy addressing different audiences and stakeholders to facilitate adoption of the injection safety recommendations according to a strategy in 2017 [13]. A package of tools (available online) promote communication for behaviour change among patients and health care workers to reduce injection overuse and ensure safety. More recent and higher quality data are needed to monitor the evolution of injection safety since 2011, including to track progress before and after the launch of the WHO policy [14]. However, systematic reviews can be costly and time consuming. The ongoing collection of injection practices information within DHS provided an opportunity to obtain new estimates easily and rapidly. The objectives were to describe injection practices since 2011, including heterogeneity by region and gender, and to determine if injection safety has improved since 2011.

## Methods

We reviewed healthcare injection practices worldwide in terms of frequency and safety according to the indicators included in DHS since 2011 and until 2015. The datasets analyzed during the current review are available in the DHS Program webpage (https://www.dhsprogram.com/). If countries surveyed since 2011 had a prior DHS survey data including injection practice up to 2010, we included the prior survey to compare data in 2004–2010 and in 2011–2015.

### Data collection during the DHS surveys

DHS questionnaires were conducted in countries and adapted from template survey instruments developed by the DHS programme to reflect the population and health issues relevant to each country. The questionnaire was translated into each native language. A nationally representative sample of households (women age 15–49 and men age 15–49, 54, or 59) was eligible for individual interviews that were performed individually. We focused on two template questions. These were ‘have you had an injection for any reason in the last 12 months? If yes, how many injections have you had?’ and ‘the last time you got an injection from a health worker, did he/she take the syringe and needle from a new, unopened package?’. When considering DHS questionnaire items, we used the term ‘frequency’ to refer to the average number of health care injections per person in the last 12 months, and used the term ‘safety’ to refer to the proportion of injections for which the respondent mentioned the syringe and needle had been taken from a new, unopened package.

### Search for DHS surveys

We accessed the homepage of DHS Program to search all DHS final reports in 2011–2015 for data on injection practices. If the surveyed year spanned over two years (such as 2015–16), we considered the survey to have been conducted in the year it was initiated. When the data was available, we also searched the survey data conducted in 2004–2010 in the same country for comparison.

### Data abstraction

We extracted the data on frequency and safety. The denominator for frequency was the total number of respondents. The denominator for safety was the number of respondents who had received at least one health care injection in the last 12 months.

### Data analysis

Some countries had only information on injections received in the last six months. In that case, we doubled frequency to adjust for the different referent period as done in prior reviews [2]. We multiplied the number of injections received by the proportion unsafe to calculate the number of unsafe injection per person per year [(the average number of health care injections per person in the last 12 months) x {1-(the proportion of injections from a new package)}]. For countries that had surveys conducted at the subnational level, we calculated the average value of the subnational surveys.

We combined the data from the various surveys to estimate the global situation in terms of frequency and safety. We compared gender groups, regions, and countries in terms of the number of unsafe injections. For countries that had more than two DHS surveys since 2004, we compared practices in 2004–2010 and in 2011–2015 in terms of safety and frequency. If there were multiple surveys up to 2010, we adopted the most recent one to reflect on the most recent evolution. For countries that only had data on women, we restricted the comparison to women for the two time periods.

## Results

### DHS surveys available

As of December 2017, we identified 92 countries with DHS data available between 2004 and 2015. Among these countries, 43% (40/92 countries) included injection safety data for 2011–2015 (Table 1). Among these, 40 countries, 40% (16/40 countries) also had the data in 2004–2010 available for comparison over time (Table 1).

**Table 1:** 40 countries with injection practices data -in DHS surveys conducted between 2011 and 2015 and 16 countries with corresponding prior data (2004–2010). As of December 2017, we identified 92 countries with DHS data available between 2004 and 2015. Among these countries, 43% (40/92 countries) included injection safety data for 2011–2015. Among these, 40 countries, 40% (16/40 countries) also had the data in 2004–2010 available for comparison over time

Region	Country	Year with data available	Year with data available
		(2011–2015)	(2004–2010)
African	Benin	2011	2006^c^
African	Cameroon	2011	No data
African	Congo	2011	No data
African	Cote d’Ivoire	2011	No data
African	Equatorial Guinea	2011	No data
African	Ethiopia	2011	2005
African	Mozambique	2011	No data
African	Uganda	2011	2006
African	Comoros	2012	No data
African	Gabon	2012	No data
African	Mali	2012	2006^c^
African	Niger	2012	No data
African	Gambia	2013	No data
African	Liberia	2013	2007
African	Namibia	2013	2006
African	Nigeria	2013	2008
African	Sierra Leone	2013	2008
African	Togo	2013	No data
African	Zambia	2013	2007
African	Chad	2014	No data
African	Ghana	2014	2008
African	Kenya	2014	No data
African	Lesotho	2014	No data
African	Rwanda	2014	2005, 2010
African	Tanzania	2015	2004, 2010^c^
African	Zimbabwe	2015	2005, 2010
Americas	Honduras^a^	2011	No data
Americas	Haiti	2012	2005
Americas	Dominican Republic	2013	No data
Eastern Mediterranean	Pakistan	2012	2006^c^
Eastern Mediterranean	Yemen	2013^c^	No data
Eastern Mediterranean	Afghanistan	2015	No data
European	Kyrgyz Republic	2012	No data
European	Tajikistan	2012^c^	No data
European	Armenia	2015	2010
South-East Asia	Nepal	2011	No data
South-East Asia	Indonesia	2012	No data
South-East Asia	India^b^	2015	No data
South-East Asia	Myanmar	2015	No data
Western Pacific	Cambodia	2014	No data

^a^ Only frequency data available (no safety data)

^b^ Average value of the five areas surveyed

^c^ Only female data available (Only female data used in the comparisons)

### Safety and frequency

Overall, respondents reported 1.64 injections per person per year (*N* = 840,711) in 2011–2015. Of these, 96.1% (*N* = 279,620) were reported to have been given with devices taken out of a new packet (i.e. were considered to be safe).

### Regional variations

**Fig. 1:**
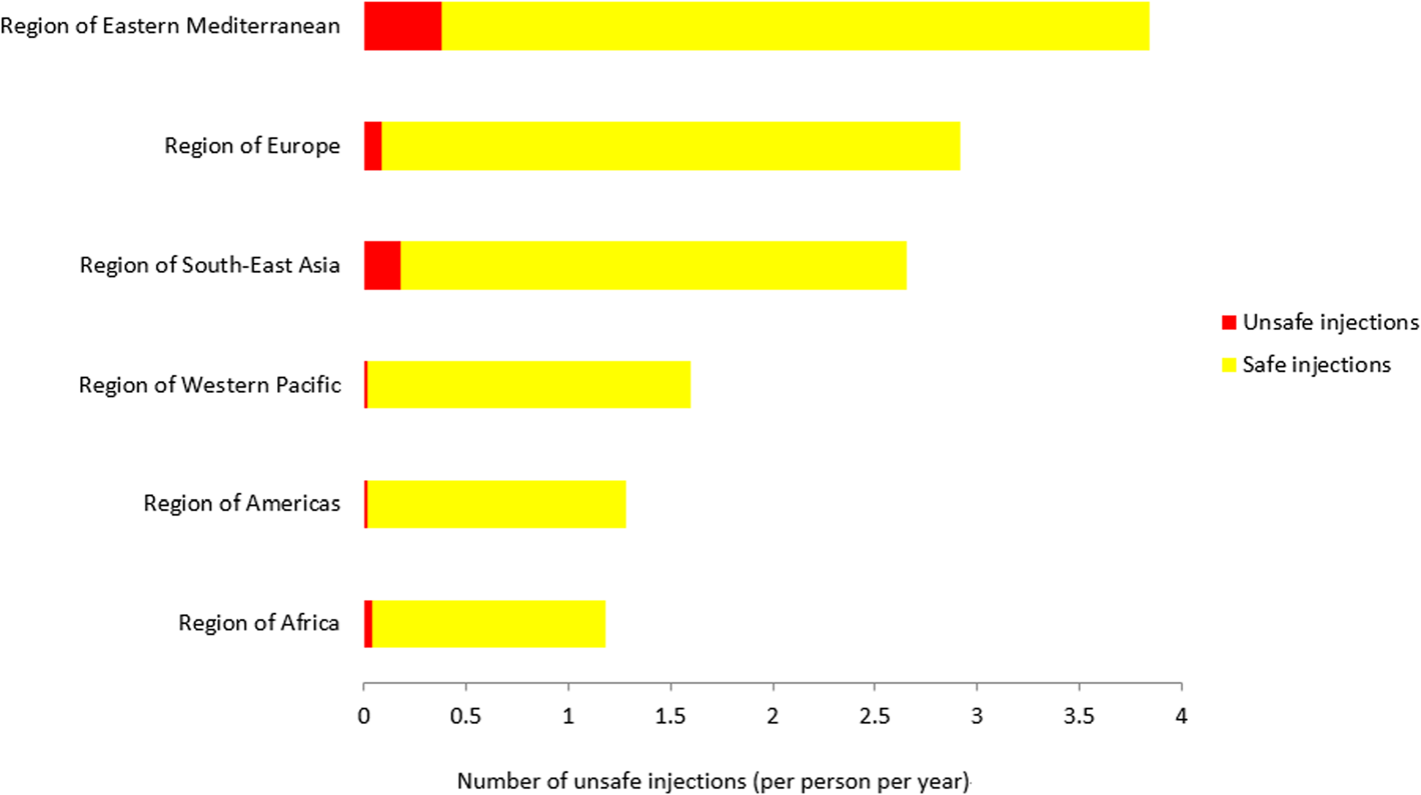
Annual number of safe and unsafe injections, by WHO region, DHS surveys conducted in 2011–2015. The Eastern Mediterranean Region had the highest number of unsafe injections (0.376 per person per year). The Eastern Mediterranean Region also had the highest average number of health care injections per person in the last 12 months and the lowest proportion of injections with devices from a new package. The Eastern Mediterranean Region was followed by the South-East Asia Region (0.178 per person per year), the European Region (0.085 per person per year), the African Region (0.039 per person per year), the region of the Americas (0.020 per person per year), and the Western Pacific Region (0.019 per person per year)

The Eastern Mediterranean Region (EMR) had the highest number of unsafe injections (0.376 per person per year). EMR also had the highest average number of health care injections per person in the last 12 months and the lowest proportion of injections with devices from a new package (Table 2 and Fig. 1). EMR was followed by the South-East Asia Region (SEAR, 0.178 per person per year), the European Region (EUR, 0.085 per person per year), the African Region (AFR, 0.039 per person per year), the region of the Americas (AMR, 0.020 per person per year), and the Western Pacific Region (WPR, 0.019 per person per year). Detailed injection practices in counties according to DHS surveys conducted in 2011–2015 is reported in Table 3.

**Table 2:** Injection practices in WHO regions according to DHS surveys conducted in 2011–2015. The Eastern Mediterranean Region had the highest number of unsafe injections (0.376 per person per year). The Eastern Mediterranean Region also had the highest average number of health care injections per person in the last 12 months and the lowest proportion of injections with devices from a new package. The Eastern Mediterranean Region was followed by the South-East Asia Region (0.178 per person per year), the European Region (0.085 per person per year), the African Region (0.039 per person per year), the region of the Americas (0.020 per person per year), and the Western Pacific Region (0.019 per person per year)

	Frequency		Safety		Number of unsafe injections per person per year ^a^
Country	Health care injections per person per year	Sample size	Use of an unopened syringe or needle	Sample size	
African	1.18	466,836	96.7%	144,115	0.039
Americas	1.28	73,335	98.4%	12,951	0.020
Eastern Mediterranean	3.84	82,347	90.2%	32,467	0.376
European	2.92	29,148	97.1%	6,881	0.085
South-East Asia	2.66	166,277	93.3%	75,233	0.178
Western Pacific	1.60	22,768	98.8%	7,973	0.019
Total	1.64	840,711	96.1%	279,620	0.064

^a^ Number of injections multiplied by the proportion unsafe

**Table 3:** Injection practices in counties according to DHS surveys conducted in 2011–2015. Detailed injection practices in counties according to DHS surveys conducted in 2011–2015. The data was divided by country from Table 2

		Frequency		Safety	
Region	Country	Health care injections per person per year	Sample size	Use of an unopened syringe or needle	Sample size
African	Benin	0.60	21,739	94.7%	3,419
African	Cameroon	1.80	14,648	97.6%	5,883
African	Congo	1.80	15,964	98.3%	4,042
African	Cote d’Ivoire	1.20	15,195	96.8%	5,767
African	Equatorial Guinea	2.60	5,400	96.1%	2,063
African	Ethiopia	1.35	30,625	97.6%	10,053
African	Mozambique	0.50	17,780	94.6%	3,196
African	Uganda	1.65	10,969	95.6%	4,328
African	Comoros	0.60	7,496	91.7%	1,486
African	Gabon	1.20	14,076	97.6%	4,626
African	Mali	0.70	14,823	98.0%	3,231
African	Niger	0.80	15,088	96.8%	5,539
African	Gambia	0.90	14,054	97.5%	4,438
African	Liberia	1.65	13,357	98.1%	5,372
African	Namibia	0.85	13,197	97.2%	4,028
African	Nigeria	1.10	56,307	97.3%	14,097
African	Sierra Leone	1.95	23,920	97.4%	9,581
African	Togo	1.15	13,956	94.5%	4,207
African	Zambia	0.75	31,184	97.0%	6,956
African	Chad	2.05	11,433	94.1%	4,124
African	Ghana	0.70	13,784	98.0%	4,030
African	Kenya	1.45	27,444	98.5%	10,877
African	Lesotho	0.75	9,552	95.2%	2,764
African	Rwanda	1.30	19,714	99.2%	10,977
African	Tanzania	0.90	16,780	97.6%	4,786
African	Zimbabwe	0.50	18,351	97.6%	4,245
Americas	Honduras^a^	1.60	29,877	No data	No data
Americas	Haiti	0.60	23,780	98.4%	5,720
Americas	Dominican Republic	1.65	19,678	98.5%	7,231
Eastern Mediterranean	Pakistan	5.15	16,692	87.7%	9,875
Eastern Mediterranean	Yemen^b^	3.30	25,434	91.8%	8,989
Eastern Mediterranean	Afghanistan	2.80	40,221	91.8%	13,603
European	Kyrgyz Republic	2.55	10,621	96.5%	2,720
European	Tajikistan^b^	7.10	9,656	98.7%	2,963
European	Armenia	1.20	8,871	97.1%	1,198
South-East Asia	Nepal	1.20	16,795	98.3%	5,430
South-East Asia	Indonesia	1.35	54,913	90.0%	23,557
South-East Asia	India^c^	5.82	76,947	86.5%	36,902
South-East Asia	Myanmar	2.25	17,622	98.7%	9,344
Western Pacific	Cambodia	1.60	22,768	98.8%	7,973

^a^ Only frequency data available

^b^ Only female data available

^c^ Average value of the five areas surveyed

### Analysis by gender

The average number of health care injections per person in the last 12 months was higher in women (1.85 per person per year, sample size: 575,490) than in men (1.41 per person per year, sample size: 265,221). However, the proportion of injection devices taken from a new package was almost the same in women (96.2%, sample size: 202,969) and in men (96.0%, sample size: 76,651). Overall, the number of unsafe injections per person and per year was 0.071 in women and 0.056 in men.

### Evolution over time

Among the 16 countries with 2004–2010 and 2011–2015 data, the average annual number of injections decreased from 1.44 (sample size: 252,513) to 1.25 (sample size: 317,886). Similarly, the average proportion of safe injection increased from 95.9% (sample size: 70,953) to 97.0% (sample size: 98,103, Table 4). In terms of frequency, of 16 countries, 9 (56%) had improved (average improvement: 0.49, range: 0.10–2.35), 1 had not changed, and 6 (38%) had deteriorated (average deterioration: 0.18, range: 0.05–0.30). In terms of safety, 11 of 16 countries (69%) improved (average improvement: 1.7%, range: 0.2–5.1%) and 5 of 16 countries (31%) deteriorated (average deterioration: 0.8%, range: 0.1–1.4%). The annual number of unsafe injections improved in 81% (13 of 16 countries) and deteriorated in 19% (3 of 16 countries). In Pakistan, the number of unsafe injections was the highest among the 16 countries. Among females for whom data was available in the two time periods. The number of unsafe injections did not change substantially between 2006 (0.71 per person per year) and 2012 (0.80 per person per year).

**Table 4:** Comparison of injections practices up to 2010 and since 2011 in the 16 countries where DHS data were available for comparison. Among the 16 countries with 2004–2010 and 2011–2015 data, the average annual number of injections decreased from 1.44 (sample size: 252,513) to 1.25 (sample size: 317,886). Similarly, the average proportion of safe injection increased from 95.9% (sample size: 70,953) to 97.0% (sample size: 98,103)

	Frequency		Safety	
	Health care injections per person per year	Sample size	Use an unopened syringe or needle	Sample size
Up to 2010^a^	1.44	252,513	95.9%	70,953
Since 2011	1.25	317,866	97.0%	98,103

^a^ Use only more recent data if there are multiple data

## Discussion

In 2010, a review of injection practices globally reported improvement in injection safety following the implementation of joint efforts from WHO, partners of the Safe Injection Global Network, other agencies and countries [10, 11]. However, it also pointed to the absence of improvement in reducing the frequency of health care injections annually. This new rapid review provides an indication of the evolution of the situation since 2011, and sheds light on the additional progress and setbacks.

The most recent DHS surveys suggested that in 2011–2015, the trend towards improvement of injection practices continued. However, injection frequency and safety continued to vary widely by region. The WHO EMR has the highest number of injections and the highest proportion of unsafe injections, followed by the WHO SEAR. The number of unsafe healthcare injection in the WHO EMR was also the highest in the world in 2000 (2.96 per person per year) [2] and in 2010 (0.57 per person per year) [10]. Many injections are administered by private health care providers who may not have formal medical qualification in these regions. In such informal situations, the attitude of the healthcare provider also promotes the abuse of injections. Compliance of standard treatment guidelines is not common. To meet the demands of the user, injections are often utilized on an “ad hoc” basis to administer drug mixtures such as antibiotics, analgesics, vitamins or antihistamines [15, 16]. If safe injections were administered, reduction of injection overuse would only be a matter of facilitating rational use of medicines [17]. However, reuse of syringes frequently leads to transmission of bloodborne pathogens [18–25]. In EMR and SEAR, especially India and Pakistan, blood-borne pathogens are prevalent and can be transmitted, around 1.7–5.6% for HBV [26], 0.5–3.8% for HCV [27], and 0.3–0.1% for HIV [28]. For instance, unsafe health care injections remain a key driver of the HCV epidemic in Pakistan [29]. It is also a possible vector of HIV infection [30]. This could be prevented as patients’ knowledge regarding transmission of bloodborne pathogens drives consumer demand for new syringes [31, 32].

According to DHS, women generally received more injections than men. The reasons for this may include obstetrical and gynecological conditions, including contraception, delivery, and prevention of maternal and neonatal tetanus. DHS data also suggest that there is no difference in injection safety between women and men. Some of the injections received by women could be received through programmes such as immunization or contraceptive treatment rather than informal health care. However, the absolute number of unsafe injections was higher in women than in men. Actually, exposure to healthcare (e.g. health care injections, hospitalizations and pregnancies) has been associated with HCV infection among women in Pakistan [33, 34]. Addressing this issue has the potential to reduce gender inequalities in injection safety. Since 1999, the principle of “bundling” has been proposed to ensure the safety of injections given through key public health programmes, so that the sources of financing of immunization and contraception commodities also pay for safe injection devices and safety boxes for disposal of used injection equipment [35]. Bundling refers to the concept of a theoretical “bundle” which comprises each of the following items: 1) good quality vaccines, 2) auto-disable syringes, and 3) safety boxes. “Bundling” does not have physical connotation and has no implication that items should be “packaged” together [36]. In EPI, application of the concept of bundling represents a substantial incremental cost as vaccines are inexpensive. However, implementation is facilitated since immunization services are usually centrally funded [37]. For therapeutic injections, the concept of bundling was generalized into broader “guiding principles for injection device security” [38]. The incremental cost is much lower because medicines are usually more expensive than the injection device required to inject them [39]. However, implementation is challenging since the funding of health care services is usually more fragmented.

Comparison of the 2004–2010 and 2011–2015 periods in 16 countries with available data suggested an improvement in many countries. Concerted efforts of ministries of health, local health facilities, non-profit organizations, and donors may explain some of the progress. However, in Pakistan, Uganda and Zambia, the number of unsafe injections did not change substantially since 2011. Pakistan is a country with a rapidly increasing population. The effects of the reduction in unsafe injections might be reduced by the population growth in some densely populated regions. However, in theory, the scale up of safe injections could be more cost effective and positively associated with infections averted in growing populations [40]. This is important because Pakistan has the second highest number of HCV infections in the world [27].

Injection-associated transmission of bloodborne pathogens can be prevented through the development of a strategy to reduce injection overuse and achieve injection safety and its implementation by a national coalition [41]. Achieving this goal necessitates the establishment of a national multidisciplinary cooperation between the Ministry of Health and stakeholders such as private healthcare providers and non-governmental organizations. Changing behavior strategies towards consumers and the public, private and general healthcare professionals should be strongly supported. To implement such a strategy, WHO suggests a package of tools available online for safe and appropriate injection use in planning, implementation and evaluation of national policies in order to support national policy makers [13]. Equipment and supplies should be provided to eradicate the re-use of syringes and needles without sterilization. Sharps waste should be managed to ensure that disposable syringes and needles are not re-used and do not lead to needlestick injuries [38]. In addition, since 2016, WHO recommends a policy to prefer the use of sharp injury protection syringes for intramuscular, intradermal and subcutaneous injections over the use of re-use prevention syringes for intramuscular, intradermal and subcutaneous injections [12]. Syringes with sharps injury prevention features reduce the incidence of needlestick injuries [42]. Healthcare workers perceive injection safety devices to be generally easy to use, safe, and tolerated by patients [43]. Rapid implementation of this policy could address residual injection safety issues in resource-limited settings where unsafe practices persist.

Our review and analysis of existing data have a number of limitations. Some limitations are inherent to the methods used by DHS. Survey respondents were asked whether they had received any injections from a health worker in last 6 or 12 months before the survey. Therefore, they might not have noticed or remembered precisely whether the injection they are receiving was given with a new needle and syringe from an unopened package when interviewed months later (recall bias). Furthermore, the respondents might tend to answer questions in a manner that will be viewed socially favorable because the data was collected by questionnaire (social desirability bias). There are also limitations due to our review methods. First, we did not calculate confidence intervals, did not perform statistical comparisons and did not model regional or global estimates. Calculation of confidence intervals in statistical analyses is based on the assumption of random sampling, yet the countries that were included in the DHS programme were not chosen at random. Further, the low proportion of unsafe injections, the lack of variability in terms of income groups and the limited sample size of countries included would have limited efforts to model regional and global estimates. Second, we used only one data source (DHS), which provides limited evidence in some areas while there could be other data sources for injection safety. Third, doubling the six month recall may overestimate the response that would’ve been generated by asking people to recall 12 months. Fourth, there are different samples sizes by the different regions or countries, therefore, it is hard to compare across regions precisely. Finally, there is large variation globally by region and a lack of data from many countries. Of 92 countries with DHS data available, injection safety data were available from only 40 countries, and these differed from the 16 countries with 2004–2010 data. Overall, our estimation is an imperfect reflection of the current status worldwide, and the review is limited to healthcare injections. In many countries non-healthcare injections, including injecting drug use, play an important role in the transmission of bloodborne infections through unsafe injections.

## Conclusion

In conclusion, despite substantial improvements in the past decade, unsafe injections persist worldwide, and continue to vary widely by WHO regions. Women continue to be at higher risk for the consequences of unsafe injections than men. Reported injection practices continued to improve in many countries, but improvements appear to have levelled off since 2011 in some countries, and the situation may have deteriorated in others. On the basis of these findings, we suggest a number of actions. First, strong promotion of the safe and appropriate use of injections is needed, particularly in regions most affected by unsafe injection practices, including the WHO Eastern Mediterranean and South-East Asia regions. Use of WHO recommendations and implementation tools can facilitate implementation [12]. Second, we must address injection safety in the field of reproductive and maternal health services to eliminate any gender inequalities concerning injection safety. Third, stringent efforts are needed to prevent injection-associated infections with bloodborne pathogens through improved technologies. A targeted approach with dissemination of auto-disable and re-use prevention syringes in resource-limited settings where safety remains an issue and the risk of injection-associated infection is high could eliminate unsafe injections. Finally, there is a need to collect high-quality data on injection safety worldwide and to make use of the available DHS data and other sources of information to justify, guide, and evaluate national safe and appropriate use of injection policies.

## Data Availability

The raw data we extracted and used for our study can be accessed by visiting the following website, https://www.dhsprogram.com/data/available-datasets.cfmAfter that, we downloaded “Demographic and Health Survey” of each country and refered to the table on “Prevalence of medical injections”.

## Abbreviations

AFR: The African Region
AMR: The region of the Americas
DHS: The Demographic and Health Surveys
EMR: The Eastern Mediterranean Region
EUR: The European Region
HBV: Hepatitis B virusHCV hepatitis C virus
HIV: Human immunodeficiency virus
SEAR: The South-East Asia Region
SIGN: The Safe Injection Global Network
WHO: World Health Organization
WPR: The Western Pacific Region
